# Macrophage activation syndrome associated with griscelli syndrome type 2: case report and review of literature

**DOI:** 10.11604/pamj.2018.29.75.12353

**Published:** 2018-01-25

**Authors:** Zakia Sefsafi, Brahim El Hasbaoui, Amina Kili, Aomar Agadr, Mohammed Khattab

**Affiliations:** 1 Center for Hematology and Oncology Paediatrics, Children’s hospital, Faculty of Medicine and Pharmacy, University Mohammed V, Rabat, Morocco; 2 Department of Pediatrics, Military Teaching Hospital Mohammed V, Faculty of Medicine and Pharmacy, University Mohammed V, Rabat, Morocco

**Keywords:** Macrophage activation syndrome, griscelli syndrome type 2, haemophagocytosis, T cells, cytokines

## Abstract

Macrophage activation syndrome (MAS) is a severe and potentially fatal life-threatening condition associated with excessive activation and expansion of T cells with macrophages and a high expression of cytokines, resulting in an uncontrolled inflammatory response, with high levels of macrophage colony-stimulating factor and causing multiorgan damage. This syndrome is classified into primary (genetic/familial) or secondary forms to several etiologies, such as infections, neoplasias mainly hemopathies or autoimmune diseases. It is characterised clinically by unremitting high fever, pancytopaenia, hepatosplenomegaly, hepatic dysfunction, encephalopathy, coagulation abnormalities and sharply increased levels of ferritin. The pathognomonic feature of the syndrome is seen on bone marrow examination, which frequently, though not always, reveals numerous morphologically benign macrophages exhibiting haemophagocytic activity. Because MAS can follow a rapidly fatal course, prompt recognition of its clinical and laboratory features and immediate therapeutic intervention are essential. However, it is difficult to distinguish underlying disease flare, infectious complications or medication side effects from MAS. Although, the pathogenesis of MAS is unclear, the hallmark of the syndrome is an uncontrolled activation and proliferation of T lymphocytes and macrophages, leading to massive hypersecretion of pro-inflammatory cytokines. Mutations in cytolytic pathway genes are increasingly being recognised in children who develop MAS in his secondary form. We present here a case of Macrophage activation syndrome associated with Griscelli syndrome type 2 in a 3-years-old boy who had been referred due to severe sepsis with non-remitting high fever, generalized lymphoadenopathy and hepato-splenomegaly. Laboratory data revealed pancytopenia with high concentrations of triglycerides, ferritin and lactic dehydrogenase while the bone marrow revealed numerous morphologically benign macrophages with haemophagocytic activity that comforting the diagnosis of a SAM according to Ravelli and HLH-2004 criteria. Griscelli syndrome (GS) was evoked on; consanguineous family, recurrent infection, very light silvery-gray color of the hair and eyebrows, Light microscopy examination of the hair showed large, irregular clumps of pigments characteristic of GS. The molecular biology showed mutation in RAB27A gene confirming the diagnosis of a Griscelli syndrome type 2. The first-line therapy was based on the parenteral administration of high doses of corticosteroids, associated with immunosuppressive drugs, cyclosporine A and etoposide waiting for bone marrow transplantation (BMT).

## Introduction

Macrophage activation syndrome (MAS) is a group of diseases characterized by a severe acute inflammatory syndrome, usually underdiagnosed. MAS can mimic several other life-threatening conditions, including sepsis, shock and multi-organ failure. Clinical features which may predict the onset of MAS include high-grade fever, hepato-splenomegaly, cytopenias, hyperferritinemia, elevated c-reactive protein (CRP) with falling erythrocyte sedimentation rate (ESR) (due to hypofibrinogenemia) and hemophagocytosis identified in the bone marrow or in other tissues of the reticuloendothelial system [1]. The mortality rate of MAS can be high, therefore, early diagnosis of MAS and prompt management may improve clinical outcomes. Unfortunately, the features of MAS overlap with signs of active underlying disease, thus making its early recognition difficult [2]. This disease is caused by proliferation and activation of T cells and macrophages, causing an inflammatory response characterized by hypersecretion of cytokines such as interferon-gamma, tumor necrosis factor alpha, interleukin (IL-1), IL-6, IL-10, IL-12, IL-18, and macrophage colony stimulating factor [2]. Secondary MAS is the result of an immunological reaction caused by autoimmune disease, infection, exposure to drugs and neoplasms [1-3]. MAS secondary to autoimmune diseases has certain differences from the other types such as very high hyperferritinemia, a decrease in erythrocyte sedimentation rate, a mild cytopenia and a more pronounced initial coagulopathy [3]. MAS is a frequent complication of Griscelli syndrome type 2 which is a fatal autosomal recessive disorder, caused by mutation in RAB27A gene [4, 5], it is associated with a primary immunodeficiency due to an impairment of T cell and natural killer cytotoxic activity, which leads to susceptibility to repeated infections and hemophagocytic syndrome or hemophagocytic lymphohistiocytosis (HLH). We report a case of Macrophage activation syndrome associated with Griscelli syndrome type 2.

## Patient and observation

We present here a case of Macrophage activation syndrome associated with Griscelli syndrome type 2 in a 3-years-old male born of consanguineous parentage. His birth histories, his familie social history and developmental milestones were unremarkable. He was born at full term with no antenatal or perinatal complications, He was on exclusively breast-fed, food diversification was started at 6 months old, his weight, length and psychomotor development were within the normal range, the child was described as a good eater, was on a normal diet and was thriving appropriately. Furthermore the boy presented a progressive abdominal distension since birth, progressive pallor and recurrent episodes of fever since 1 year of age. There was history of blood transfusions for last 2 months. On the other side there was no history of jaundice, vomiting, urinary or bowel complaints, bleeding from any site or neurological complaints. On admission, he was very pale with silvery gray scalp hair, white eyelashes, he was hypotonic, tachycardic, fever of 40°C, lymphoadenopathy and hepato-splenomegaly. The blood count showed pancytopenia; 1980 white blood cells/µL (VN 4000-13 500/µL), neutrophils 820/µL, 910 cells/µL, hemoglobin (Hb) 6.3 g/dL (11.5-14 VN, 5 g/dL), platelets 44 000/µL (VN 150 000-400 000/µL), There were no giant cytoplasmic granules in leucocytes. The liver function tests were normal expect low albumin (2.1 mg/dL) and increased alkaline phosphatase (1196 mg/dL) with low fibrinogen (1.7g/l). Serum triglycerides, ferrintin and lactic dehydrogenase were very high; respectively 4.75g/L; 2763µg/L and 597U/L. the C reactive protein (CRP) was elevated at 83mg/l.

**Figure 1:**
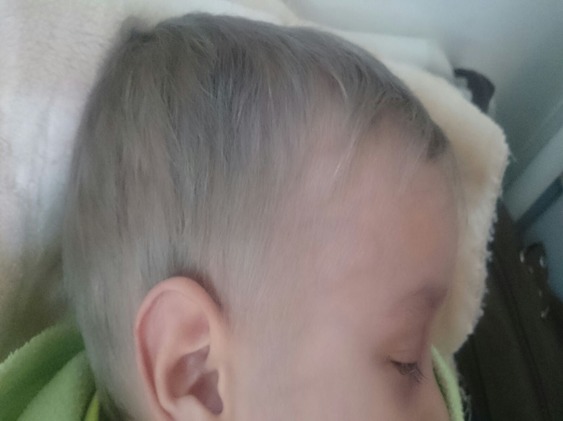
Picture of our patient showing silvery hairs, eyelashes and eyebrows

**Figure 2:**
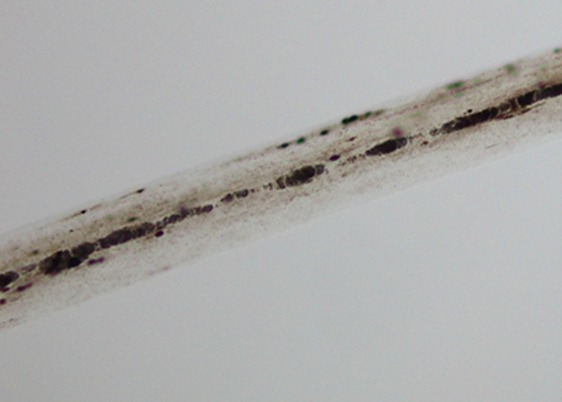
Light microscopy examination of the hair of our patient showing a large, irregular

A chest X-ray was normal while the abdominal ultrasound showed a hepatomegaly and a splenomegaly, free biliary ducts without ascites. Because of hepato-splenomegaly, pancytopenia, hyperferritinemia and hypofibrinogenemia the diagnosis of Macrophage activation syndrome was evoked indicating a bone marrow aspiration that showed numerous morphologically benign macrophages with haemophagocytic activity that comforting the diagnosis of a SAM according to Ravelli and HLH-2004 criteria Table 1. Blood culture, urine examination, malarial serology, Kala Azar serology, viral serologies such as EBV, hepatitis A, B, C and human immunodeficiency virus HIV, serologies of rickettsia, syphilis and toxoplasmosa were negatives. Serologies of CMV and rubella showed an old immunization. The immunological tests such as antinuclear antibodies (ANA), anti-LKM1, anti-mitochondria and anti-smooth muscle antibodies were negatives. Because of the consanguineous family, notion of recurrent infection and the presence of silvery-gray color of the hair and eyebrows (Figure 1), diagnosis of Griscelli Syndrome was evoked directing a light microscopy examination of the hair that showed a large, irregular clumps of pigments (Figure 2) characteristic of Griscelli Syndrome. The molecular biology showed mutation in RAB27A gene confirming the diagnosis of a Griscelli syndrome type 2. The first-line therapy in management of Macrophage activation syndrome complicating Griscelli syndrome type 2 was based on the parenteral administration of high doses of corticosteroids (methylprednisolone pulses 1g/0.73m^2^/day for 3 days then 60mg/m^2^) with poor response; persisting with involvement of the 3 cell lines in the blood count, elevated ferritin and triglycerides. Given the lack of response to steroids, Etoposide was started for 3 days associated with cyclophosphamide, the evolution was good with improvement of count blood cells, ferritin was lessening while the bone marrow showed decreased numerous of macrophages with low haemophagocytic activity. On the other side the infectious process was good managed by perfusion of Antibiotics such as Ceftazidim in combination with aminoside. The patient was sent home with prednisone and cyclosporine, He was placed on the list of bone marrow transplantation (BMT).

**Table 1:** Proposed criteria or features useful in the diagnosis of MAS

HLH-2004 criteria	Ravelli criteria	MAS Study Group
1. A molecular diagnosis consistent with HLH (i.e., reported mutations found in either PRF1 or MUNC13-4, STX11, STXBP2, Rab27a, SH2D1A, or BIRC4) OR	Laboratory criteria: Decreased platelet count (≤ 262 ×10^9^/L) Elevated aspartate aminotransferase	Falling platelet count Hyperferritinemia Evidence of macrophagehemophagocytosis in the bone marrow
2. At least five of the eight diagnostic criteria for HLH listed below Persistent fever	(>59U/L) Decreased white blood count (≤ 4.0×10^9^/L)	Increased liver enzymes Falling leukocyte count Persistent continuous
Splenomegaly	Hypofibrinogenemia (≤ 2.5 g/L)	fever >38°C
Cytopenias (affecting ≥2 of three lineages in the peripheral blood): Hemoglobin < 90 g/L, Platelets < 100 × 109/L, Neutrophils < 1.0 109/L	Clinical criteria: Central nervous systemic	Falling ESR Hypofibrinogenemia Hypertriglyceridemia
Hypertriglyceridemia (fasting triglycerides >3.0 mmol/L) and/or hypofibrinogenemia (≤ 1.5 g/L)	dysfunction (irritability, disorientation, lethargy, headache, seizures, coma)	
Hemophagocytosis in bone marrow or spleen or lymph nodes, no evidence of malignancy Serum ferritin ≥500 µg/L	Hemorrhages (purpura, easy bruising, mucosal bleeding) Hepatomegaly (≥ 3 cm below the costal margin)	Most frequent features identified by responding clinicians
Low or absent NK cell activity (according to local laboratory reference) Increased serum sIL-2R a (according to local laboratory reference)	Two or more laboratory criteria OR any two or more clinical and/or laboratory criteria	
Criteria 1 OR 2 fulfilled		

## Discussion

Griscelli syndrome (GS) is a fatal autosomal recessive disorder, first described by Griscelli et al [6] as partial albinism associated with immunodeficiency. GS was classified into three different subtypes, all of which show similar pigment dilution. In addition to pigment problems, type 1 is associated with severe primary neurological impairment such as developmental delay and mental retardation [7]. The third type is restricted to hypopigmentation defects [8]. The second type (GS2), caused by mutation in RAB27A gene [4, 5], is associated with a primary immunodeficiency due to an impairment of T cell and natural killer cytotoxic activity, which leads to susceptibility to repeated infections and hemophagocytic syndrome or hemophagocytic lymphohistiocytosis (HLH). Macrophage activation syndrome (MAS), is a life-threatening complication that refers to reactive hemophagocytic lymphohistiocytosis (HLH), which may be classified into primary, the genetic forms and secondary, the reactive forms, which are associated with infective, autoimmune, or neoplasia-related diseases [9]. Although MAS may occur at any age, it must be pointed out that multiple lines of evidence derived from pediatric patients; in fact MAS may be the most severe complication during the course of systemic onset juvenile idiopathic arthritis (SOJIA) with frequency ranging from 10% to 40% of all cases [10].

Available literature suggests that MAS may affect from 10 to 25% of AOSD patients and from 0.9 to 4.6% of systemic lupus erythematosus (SLE) patients, directly occurring from the beginning, during the course of the disease as well as triggered by infective agents particularly viral infections [2] and its high mortality rate may be dramatically influenced by an early diagnosis with consequent aggressive treatments, which have been shown to improve the survival of these patients [9]. The term MAS was proposed in 1993 by Hadchouel et al, who found evidence of activation of the monocyte-macrophage system in patients with the syndrome and noted that its clinical features were very similar to those observed in other haemophagocytic syndromes that are collectively referred to as haemophagocytic lymphohistiocytosis (HLH) [11]. Recently, the recognition that MAS belongs to HLH has led to a proposal to rename MAS according to the contemporary classifications of HLH [12]. Currently, MAS is classified among the secondary, or acquired, forms of HLH. The clinical presentation of MAS is generally acute and occasionally dramatic, requiring the admission of the patient to the intensive care unit. The onset of the syndrome is usually heralded by the sudden occurrence of non-remitting high fever, pancytopenia; as a result, patients may have purpura, easy bruising and mucosal bleeding. Liver enlargement, generalized lymphoadenopathy and increase in serum liver enzymes. There is often an alterations in coagulation tests, with prolongation of prothrombin and partial thromboplastin times, hypofibrinogenaemia, High concentrations of triglycerides and lactic dehydrogenase and low sodium levels are observed frequently. Table 1 show a proposed criteria or features useful in the diagnosis of MAS [13-15].

The acute phase of MAS is usually marked by a sharp rise of ferritin, often above 5000-10,000 ng/mL it has been suggested that measurement of serum ferritin level may assist in the diagnosis of MAS and is a useful indicator of disease activity, therapy response and prognosis. The pathognomonic feature of the syndrome is seen on bone marrow examination, which reveals numerous morphologically benign macrophages exhibiting haemophagocytic activity. MAS is a severe condition that can pursue a rapidly fatal course, prompt recognition of its clinical and laboratory features and immediate therapeutic intervention are critical. However, because it lacks a single distinguishing manifestation and is clinically heterogeneous, early diagnosis can be difficult. The diagnostic challenges posed by MAS in secondary form are compounded as it may mimic a sepsis-like syndrome or a flare of the underlying disease. The pathogenesis of MAS is still poorly understood. The starting point for pathogenetic studies in MAS is based on its close resemblance to other forms of HLH [16]. The best known of these is familial HLH (FHLH), which is a group of rare autosomal recessive immune disorders resulting from homozygous deficiency in cytolytic pathway proteins [13]. In FHLH, the uncontrolled expansion of T cells and macrophages has been related to decreased NK cell and cytotoxic T cell function [17]. Due to mutations in a variety of genes whose products are involved in the cytolytic pathway.

The cytotoxic activity of these cells is mediated by the release of cytotoxic granules that contain particular proteins, including perforin and granzymes. These mutations cause a severe impairment of cytotoxic function of NK cells and cytolytic T lymphocytes which leads to the multiple organ failure. As far as the therapy of MAS is concerned, we have to point out that, due to its rarity, specific guidelines have not been still established and anecdotally, the pivotal therapeutic strategy is the administration of high-dosage steroids. The goal of treatment is to stop the inflammatory process. The mainstay of the therapy is traditionally based on the parenteral administration of high doses of corticosteroids. However, some fatalities have been reported, even among patients treated with massive doses of corticosteroids [18]. In the mid-90s, the use of cyclosporine A (CSA) was considered, based on its proven benefit in the management of FHLH [19]. The CSA was found to be effective in some cases of MAS refractory to corticosteroids. The demonstration of the distinctive efficacy of CSA has led to propose the use of this medication as first line treatment in MAS occurring in childhood systemic inflammatory disorders [10]. The administration of high-dose intravenous immunoglobulins, cyclophosphamide, plasma-exchange and etoposide has provided conflicting results. Etoposide is part of the protocol developed for treating FHLH [13]. Biological treatments are used when steroids and/or synthetic immunosuppressive drugs have not been effective. Receptor inhibitors of TNF, IL-1 and IL-6 are an option; these should be used with caution and monitoring of side effects or complications such as infections. Rituximab has proven effective in patients with MAS secondary to systemic lupus erythematosus. Anakinra is effective in MAS secondary to systemic juvenile idiopathic arthritis; it also shows an advantage over other biological treatments.

## Conclusion

Macrophage activation syndrome (MAS) is a rare, life-threatening disease in which early diagnosis and aggressive therapeutic strategy may improve the outcome. Due to its rarity, epidemiologic data are still lacking. Hyperferritinemia is frequently associated with MAS and might modulate the cytokine storm, which is involved in the development of multiple organ failure. There is great need for new and specific therapies for this condition. Cytokine-directed therapies have the potential to target the effector cells of MAS without the myelosuppressive side effects of current therapies such as etoposide. In this regard, an intriguing target is IFN_gamma_.

## Competing interests

The authors declare no conflicts of interest.
